# Formal Modeling and Analysis of the MAL-Associated Biological Regulatory Network: Insight into Cerebral Malaria

**DOI:** 10.1371/journal.pone.0033532

**Published:** 2012-03-30

**Authors:** Jamil Ahmad, Umar Niazi, Sajid Mansoor, Umair Siddique, Jaclyn Bibby

**Affiliations:** 1 Research Centre for Modeling and Simulation (RCMS), National University of Sciences and Technology (NUST), Islamabad, Pakistan; 2 NUST Centre for Virology and Immunology (NCVI), National University of Sciences and Technology (NUST), Islamabad, Pakistan; 3 Department of Structural and Chemical Biology, University of Liverpool, Liverpool, United Kingdom; Niels Bohr Institute, Denmark

## Abstract

The discrete modeling formalism of René Thomas is a well known approach for the modeling and analysis of Biological Regulatory Networks (BRNs). This formalism uses a set of parameters which reflect the dynamics of the BRN under study. These parameters are initially unknown but may be deduced from the appropriately chosen observed dynamics of a BRN. The discrete model can be further enriched by using the model checking tool HyTech along with delay parameters. This paves the way to accurately analyse a BRN and to make predictions about critical trajectories which lead to a normal or diseased response. In this paper, we apply the formal discrete and hybrid (discrete and continuous) modeling approaches to characterize behavior of the BRN associated with MyD88-adapter-like (MAL) – a key protein involved with innate immune response to infections. In order to demonstrate the practical effectiveness of our current work, different trajectories and corresponding conditions that may lead to the development of cerebral malaria (CM) are identified. Our results suggest that the system converges towards hyperinflammation if Bruton's tyrosine kinase (BTK) remains constitutively active along with pre-existing high cytokine levels which may play an important role in CM pathogenesis.

## Introduction

Severe forms of malaria claim a huge number of lives worldwide, contributing to over a million deaths annually, mostly that of children [1]. Malaria is vectored by Anopheles mosquitoes and is a common infectious disease caused by Plasmodium parasites that readily infect blood erythrocytes [2]. In a few cases a severe pathogenesis occurs due to hyperinflammation, usually following Plasmodium falciparum infection, that may turn fatal. The blood flow through small blood vessels to the brain is severely hampered as the infected erythrocytes are sequestered by parasites causing ischaemic hypoxia and increased nitric oxide production in brain tissues, leading to coma, a condition known as diffuse encephalopathy or CM [3]–[5]. Clinical prognosis depends on factors ranging from patient's social conditions to recurrent parasitic exposure, however current evidence strongly suggests that the genetics of an individual may play an even more significant role [6]–[8].

The human innate immune system is the first line of defence against such infections and the responses include inflammation which helps to control the infection and promotes healing. Yet if left unchecked, this advantageous inflammatory response turns astray, causing effects ranging from mild allergies to severe inflammatory disorders [9]. Acute inflammatory response is initiated following an infection through the production of proinflammatory cytokines, such as the tumor necrosis factor alpha (TNF-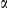) and the interferon gamma (INF-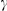) that play a prominent role in parasite destruction. The generation of inflammation is tightly regulated at multiple levels to control this production. However, in cases where pathogenesis becomes severe, chronic over production of cytokines contributes to elevated levels of a cellular messenger, induced nitric oxide synthase (iNOS). These elevated levels of iNOS plus the hypoxia caused by the parasites work in sync to create a condition of chronic hyperinflammation causing an augmentation of CM pathogenesis [3], [10].

**Figure 1:**
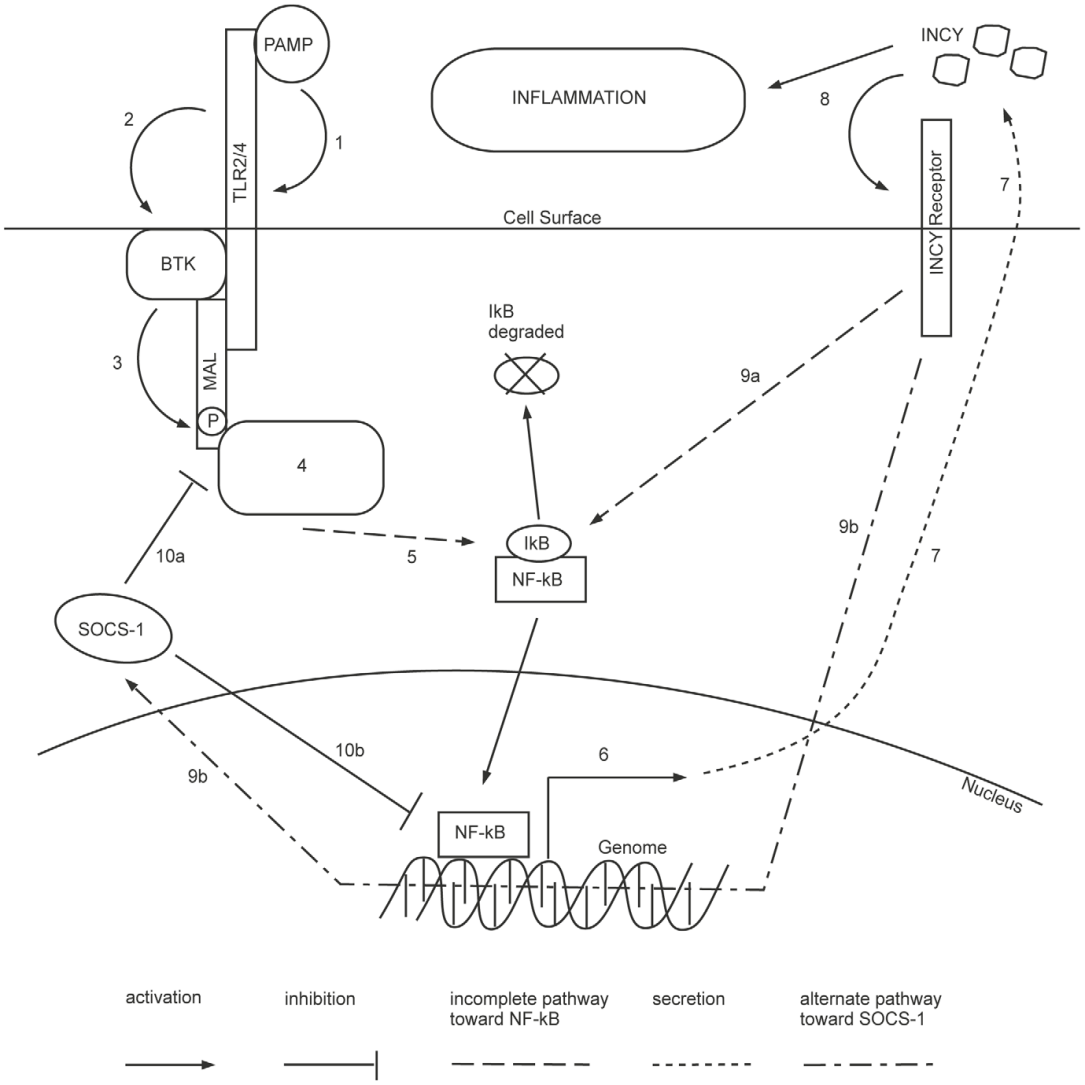
TLR2/4 signalling pathway. The TLR2/4 signalling pathway starts with recognition (1) of PAMPs by TLRs. This activates (2) BTK which phosphorylates (3) MAL. MyD88 adapter protein and kinases are recruited (4) and activated around MAL. This eventually leads to the activation (5) of NF-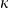
B as I
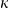
B is degraded. The proinflammatory cytokine genes are expressed (6) producing INCY that are secreted (7). INCY are responsible (8) for the production of inflammation and activation of their respective receptors. This again activates NF-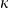 B (9a) and through an alternate pathway induces the production of SOCS-1 (9b). SOCS-1 negatively regulates MAL by polyubiquitination (10a) and blocks NF-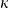 B mediated expression (10b). Abbreviations: TLR, toll like receptors; PAMPs, pathogen associated molecular patterns; BTK, bruton's tyrosine kinase; MAL, MyD88 adapter like; MyD88, myeloid differentiation response protein; NF-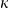
B, nuclear factor kappa-light-chain-enhancer of activated B cells; I
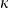
B, inhibitor of 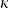
B; INCY, proinflammatory cytokines; SOCS-1, suppressor of cytokine signaling-1 [15], [24], [25].

The signal transduction pathway involved in systemic production of proinflammatory cytokines in case of malaria is initiated following the activation of the Toll like receptor 2 (TLR2) and TLR4 when they recognize glycosylphosphatidylinositols (GPIs) anchored on plasmodium membrane proteins [11]–[13]. TLRs are characteristic type I transmembrane pattern recognition receptors, used by the innate immune system to recognize conserved microbial structures or pathogen-associated molecular patterns (PAMPs). They have a conserved cytoplasmic toll-interleukin 1 receptor (TIR) domain and are included in the interleukin 1 receptor (IL-1R)/TLR super-family [14]. After stimulation by PAMPs, TLRs form dimers and begin an intricate multifaceted signalling cascade, initiated by the recruitment of adapter proteins at their cytoplasmic TIR domain. The myeloid differentiation primary response protein (MyD88) and a number of kinases (like interleukin-1 receptor-associated kinases; IRAKs) are recruited downstream. The signal transduction culminates with the activation of nuclear factor kappa-light-chain-enhancer of activated B cells (NF-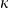
B), resulting in the expression of proinflammatory cytokine genes (see Figure 1) . For details of the TLR signalling see reference [15].

Khor et al. and others [8], [16]–[18] indicate the toll-interleukin 1 receptor domain containing adapter protein (TIRAP), also known as MyD88-adapter-like (MAL), as a key adapter protein associated with disease pathogenesis leading to hyperinflammation. They argue on the basis of single nucleotide polymorphism (SNP) analysis that wild-type MAL may lead to chronic hyperinflammation, increasing host susceptibility towards developing CM; while mutants may confer a protective effect from CM pathogenesis for its carrier host by controlling the amount of inflammation produced. Some studies have suggested MAL as an important therapeutic target for the management of CM pathogenesis [19]–[21].

Signalling regulators associated with MAL include Bruton's tyrosine kinase (BTK) which is a member of the Tec family of kinases. It acts as a positive regulator of MAL by activating it through phosphorylation [22], [23]. The inflammatory cytokines (INCY) produced not only generate inflammation but also induce SOCS-1 (suppressor of cytokine signaling-1), which degrades phosphorylated MAL through polyubiquitination. It is also known to block NF-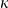
B mediated expression, thereby functioning as a negative regulator for this signalling pathway [24], [25].

**Figure 2:**
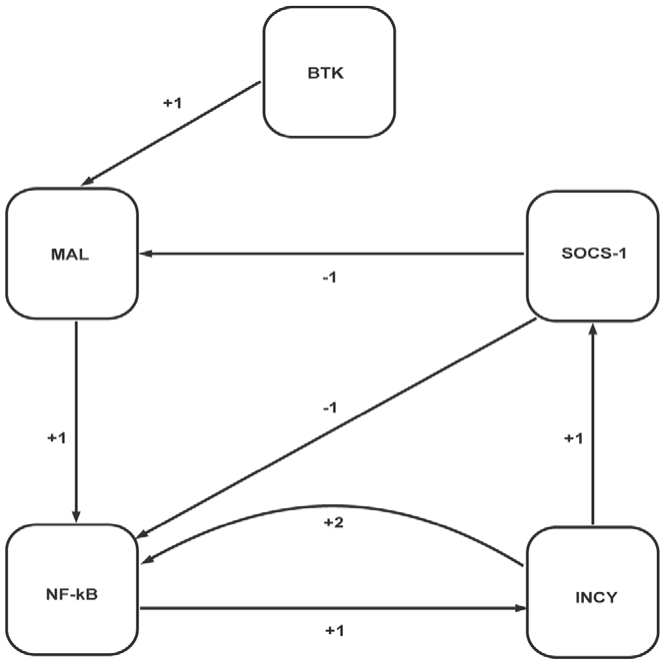
MAL associated BRN. Numerals (1 and 2) represent the threshold levels of interactions; plus (+) signs indicate activation while inactivation is indicated by a minus (−) sign. Arrows indicate the direction of activation/inactivation. The thresholds values are set according to Definition 2.

A biological regulatory network (BRN) is a set of interactions (activation and inhibition) between biological entities (e.g., proteins in a biological signalling network). The MAL associated BRN (Figure 2) was abstracted from the TLR2/4 signalling pathway (Figure 1) and includes: BTK that activates MAL through phosphorylation; SOCS-1 that degrades phosphorylated MAL by polyubiquitination; NF-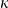
B which is the key transcription factor for initiating the expression of proinflammatory genes; and the INCY which produce the inflammation.

Two tools, GENOTECH [26] and HyTech [27], were used for the modeling and analysis of this BRN. In GENOTECH tool the qualitative modeling formalism of René Thomas [28]–[36] has been implemented. A BRN in GENOTECH is represented by a directed graph where nodes represent biological entities and edges represent interactions among them. The edges are labeled with integers representing concentration thresholds and signs of interactions (+for activation and −for inhibition). Each entity is assigned a set of logical parameters to generate a state graph (qualitative model) reflecting the possible steady state behaviors of the BRN. GENOTECH also generates a hybrid model by incorporating clocks that measure activation and degradation delays. This hybrid model is analyzed by HyTech which is a tool for the analysis of hybrid systems [37] to compute parametric constraints for the existence of a particular behavior.

This study uses qualitative discrete and hybrid modeling with delays to model the MAL associated BRN. The model characterizes conditions when the system may converge toward hyperinflammation. Initially, a discrete qualitative modeling of the BRN was done to predict qualitative behaviors or states which either lead to a normal (normal inflammatory) or diseased (hyperinflammatory) state. The qualitative model was then refined with clocks measuring regulation delays to obtain a hybrid model incorporating discrete and continuous behaviors. Our results suggest that the system converges towards hyperinflammation when concentration of cytokines is at high level. Furthermore, we obtained the conditions for each behavior of the MAL associated BRN in terms of delay constraints in order to select consistent models regarding delays (see HyTech results for delay constraints). These results give useful information such as, which variable is evolving with high production or degradation rate in order to follow a particular behavior or path. The delay constraints further suggest that for some given set of delay values, the behaviors (see Discussion section) would be very stable and for other set of values they would not exist.

In summary, we make use of qualitative in-silico modeling; a kind of computational method [38] used in systems biology, to characterize the behaviors of MAL associated BRN. At the same time we follow the trend in systems biology [39] to augment the descriptive and analytical power of models in systems biology by formal methods.

## Results

This section presents the results obtained by using the modeling and analysis tools for the MAL associated BRN (Figure 2). The first subsection describes the results obtained by the qualitative modeling and analysis of the BRN using the GENOTECH tool [26]. The results obtained by using the HyTech [27] tool for the hybrid modeling and analysis of the BRN are given in the second subsection.

### The State Graph of the MAL associated BRN

**Figure 3:**
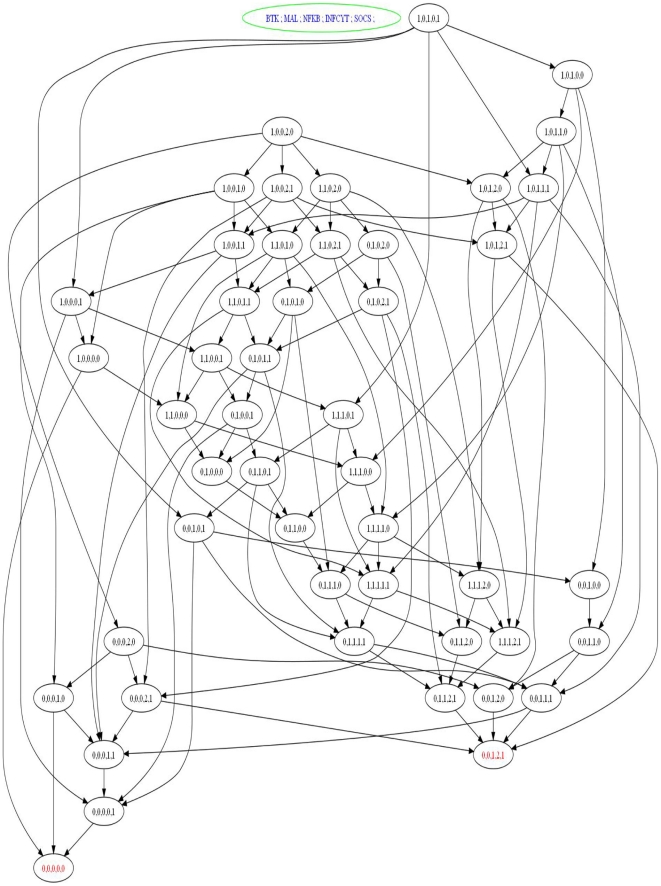
State graph of the MAL associated BRN. The complete state graph is obtained by using the GENOTECH tool. Definitions 2 and 4 assist in setting the values of the K-parameters. The K-parameters are set such that they result in a model coherent with the observed steady states behaviors. In the case of MAL-associated BRN these states are (0,0,0,0,0) and (0,0,1,2,1). In the state (0,0,0,0,0) the system does nothing when there is no signal of pathogen or returns to this state after proper response. In the state (0,0,1,2,1), the system produces inflammation continuously. The set of logical parameters is: 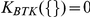, 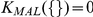, 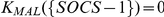, 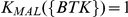, 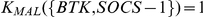, 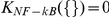, 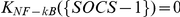, 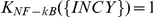, 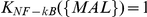, 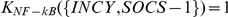, 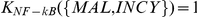, 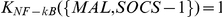, 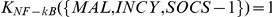, 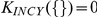, 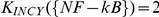, 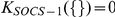 and 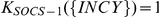.

GENOTECH is a tool for the qualitative (discrete) modeling of BRNs according to Thomas' formalism (see methods). It takes a BRN and corresponding logical parameters from a user in a simple graphical user interface (GUI) and then automatically generates the state graph in which stable states, cycles and paths between any two states can be identified. GENOTECH generates the state graph (qualitative model) of the MAL associated BRN (Figure 2) representing all possible transitions from one state to the other (Figure 3), where each state indicates the concentrations of every entity at a particular time. A state is represented by BTK, MAL, NF-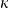
B, INCY and SOCS-1, in the respective order. In a stable state, the whole system converges, halts and cannot proceed to any other state. The stable states are called sinks and the network from any initial state either moves towards these sinks, or ends up in a cyclic path if there is a cycle in the network. From any state other than the stable states, a path that leads to and culminates at a sink is called a trajectory; and all trajectories culminate at some sink or remain in a cycle. It is an inherent property of any system to attain stability; therefore whenever the system is perturbed from its stable state or sink, it tends to shift back or reach another stable state. For the functional and realistic model of MAL-associated BRN, the generation of a specific stable state where every component has a zero concentration is imperative as this state represents the system before any perturbation ( also called the virgin state).

**Figure 4:**
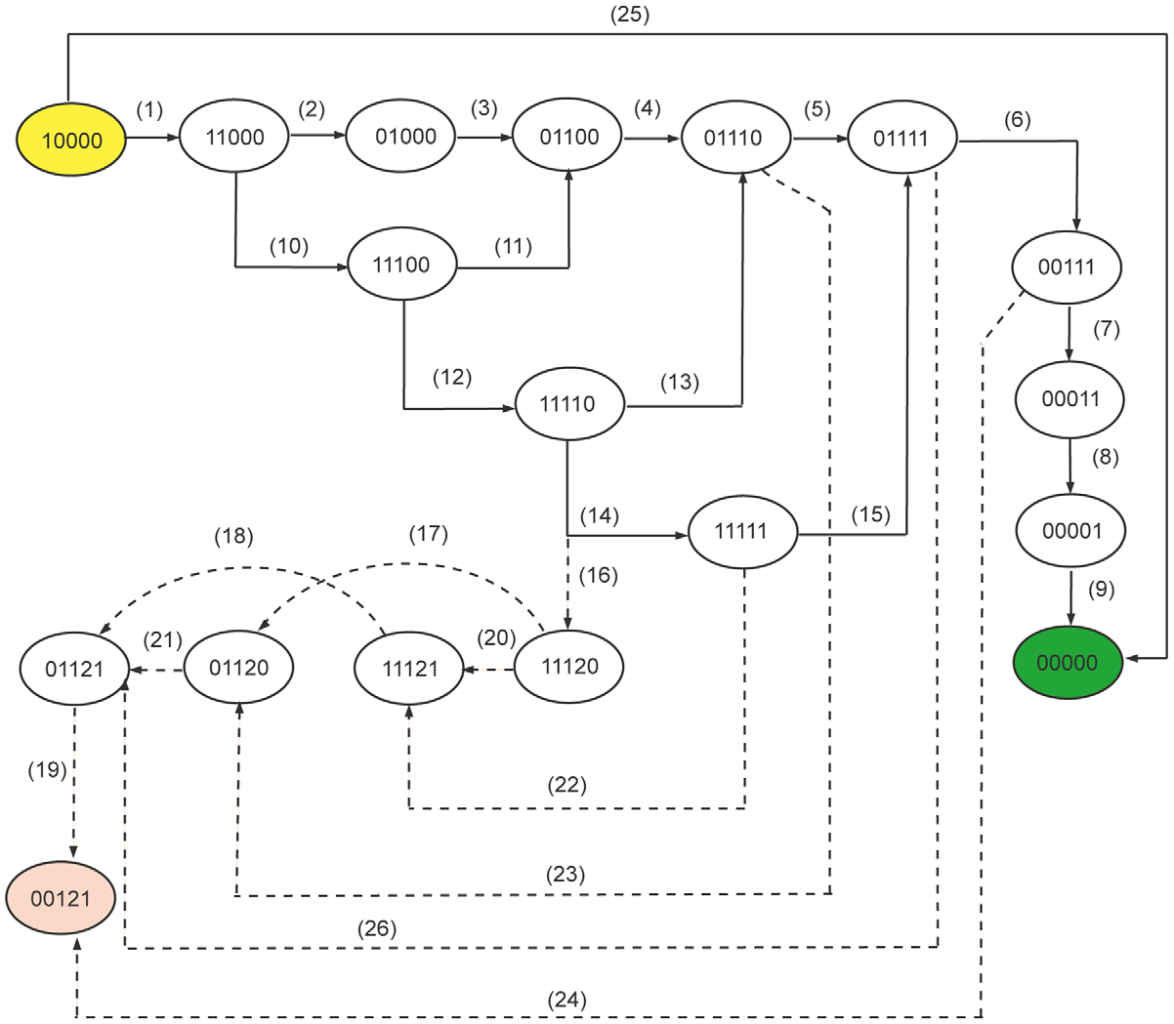
Normal and Divergent paths. Each circle represents a particular state (configuration) and inside the circle the values 0,1 and 2 represent qualitative levels of proteins according to the order (BTK, MAL, NF-kB, INCY, SOCS-1). The solid lines show the transition which leads to normal state (0,0,0,0,0) and the dotted lines show the transitions towards the state of hyperinflammation (0,0,1,2,1). The conditions for all the transitions in this figure are given in Table 1.

In our case two stable states (also called stable steady states or sinks) are produced, indicating zero concentrations for every component 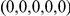 in the first state; and zero concentration for BTK and MAL, 1 for NF-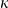
B, 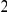 for INCY and 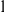 for SOCS-1 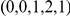 in the second state. The stable state 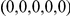 represents the normal behavior where infection is cleared whereas 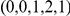 represents the diseased behavior leading to hyperinflammation. Since BTK is the initiator for this BRN, the state 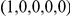 was taken as the starting state. All possible trajectories from this starting state leading to the normal and diseased states were identified (Figure 4). The trajectories leading to the sink 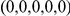 are representatives of a normal behavior against infection, where inflammation is produced to clear the infection and diminished afterwards.

### HyTech Results for Delay Constraints

All possible transitions of normal (towards (0,0,0,0,0)) and divergent (towards (0,0,1,2,1) pathways are highlighted in Figure 4 and the corresponding conditions are listed in Table 1. The state (1,0,0,0,0) is the initial state of the hybrid model. There are two possible outer transitions which are towards (0,0,0,0,0) and (1,1,0,0,0) from the initial state. The constraint 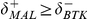 shows that if the deactivation (or degradation) delay of BTK is less than or equal to the activation delay of MAL, then the next location of the system will be (0,0,0,0,0), which is a stable state representing normal behavior. The transition 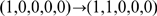 (Figure 4 and Table 1 - Transition 1) can only occur if the positive delay of MAL (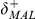) is less than or equal to the degradation delay of BTK (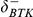), i.e. if the rate of activation of MAL is faster than the rate of degradation of BTK. The transition 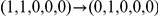 can only occur if the sum of the activation delays of MAL and NF-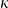
B will be greater than or equal to the degradation delay of BTK (see transition 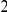 in Table 1). The transition 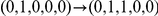 will occur unconditionally because there is only one outer transition from state (0,1,0,0,0) and this will occur after some delay 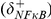 which is needed to activate NF
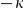
B. The transitions which lead to divergence from normal pathways (shown as dotted lines in Figure 4) are important because these can provide useful information about the BRN dynamics. The state (0,1,1,1,0) has two transitions, one towards a normal and the other towards a divergent path (see Figure 4, transitions 5 and 15). The transition 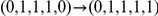 is only possible if SOCS-1 becomes active before the cytokines reaches level 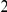 (INCY2). If this is not the case, then the network will be in state (0,1,1,2,0), which ultimately leads to the diseased state (0,0,1,2,1). Another very sensitive transition is 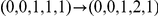, which directly leads into a diseased state due to the high level of cytokines (level 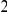). In order to follow a particular path all conditions (see Table 1) of individual transitions must be satisfied. The conditions for all paths leading to the stable states are given in Table 1 and 2 respectively. All the constraints of Table 1 and 2 were computed in 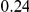 seconds on Intel Core i7 machine.

**Table 1:** Delay constraints for transitions.

Transition number	Transitions	Conditions for the transitions
1	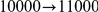	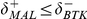
2	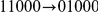	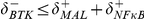
3	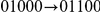	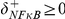
4	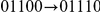	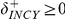
5	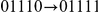	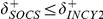
6	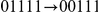	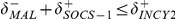
7	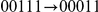	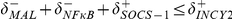
8	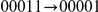	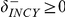
9	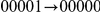	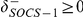
10	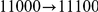	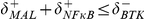
11	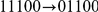	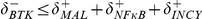
12	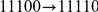	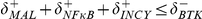
13	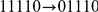	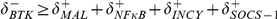
14	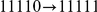	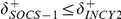
15	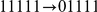	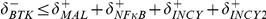
16	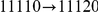	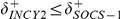
17	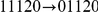	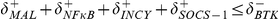
18	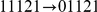	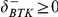
19	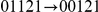	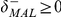
20	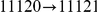	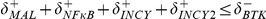
21	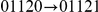	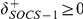
22	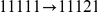	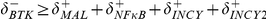
23	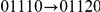	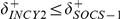
24	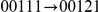	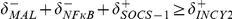
25	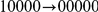	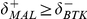
26	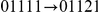	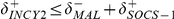

The delay constraints of all paths starting from state (10000) are automatically generated by HyTech. Constraints related to paths identified (Figure 10) are arranged in tabular form as shown in Table 2. 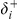 (resp. 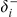) is the delay for the evolution of a protein 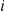 from level 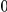 to 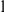 (resp. from level 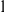 to 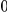). Similarly, 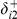 (resp. 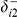) is the delay for the evolution of a protein 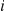 from level 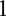 to 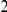 (resp. from level 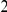 to 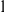).

**Table 2:** Delay constraints for paths.

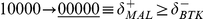
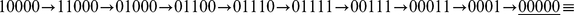
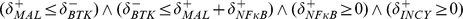
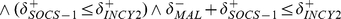
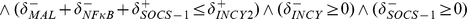
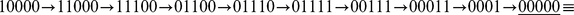
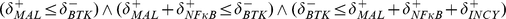
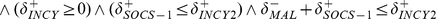
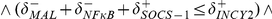
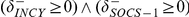
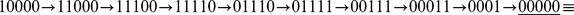
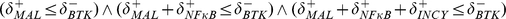
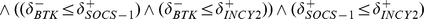
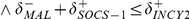
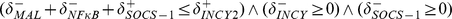
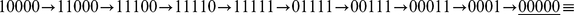
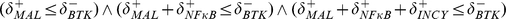
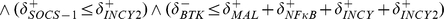
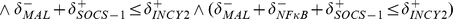
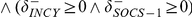
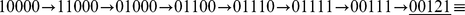
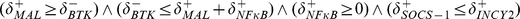
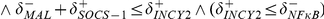
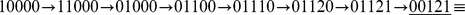
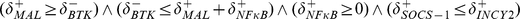
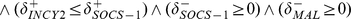
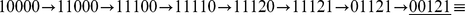
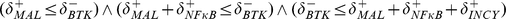
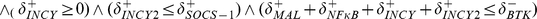
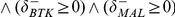
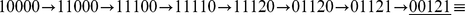
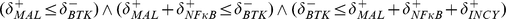
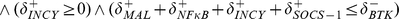
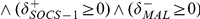
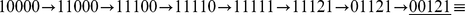
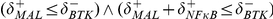
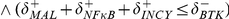
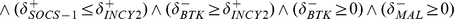

Delay constraints for the normal and diverging paths of Figure 10. All paths ending with the state (0000) are normal while others ending with (00121) are divergent. The symbol 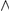 is the conjunction operator. 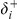 (resp. 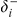) is the delay for the evolution of a protein 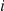 from level 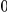 to 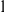 (resp. from level 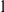 to 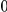). Similarly, 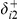 (resp. 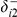) is the delay for the evolution of a protein 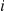 from level 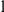 to 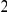 (resp. from level 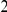 to 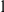).

## Discussion

MAL is imperative for TLR2/4 downstream signaling, acting both as an adapter sorter by bringing in other adapter proteins to the TLR cytoplasmic TIR domain. It also acts as a site for the assembly of several kinases important for downstream signal transduction leading to inflammation. Although production of inflammation in response to infection is not just limited to the TLR pathway, studies by Khor et al. and others have indicated a putative role of MAL in the pathogenesis of CM due to hyperinflammation [8], [16]–[18]. In this study we use discrete qualitative and hybrid modeling with delay constraints to characterize the behavior of MAL associated BRN.

### Discrete Modeling

A qualitative discrete model based on the kinetic logic of Thomas is obtained using GENOTECH by providing it information of component interactions and their respective threshold concentration levels. The GENOTECH results are rendered as a state transition graph (Figure 3) referred to as the MAL state space from here on and it represents different configurations the system may reside in as an inflammatory response is initiated. The two stable states or sinks generated indicate two distinct behaviors for the system, parallel to the normal and diseased behaviors, and any configuration of the system may eventually move toward either of these two sinks through specific trajectories or paths (Figure 3 and 4). These two states are (0,0,0,0,0), indicating zero concentration for every component; and (0,0,1,2,1), indicating zero concentration for BTK and MAL, one for NF-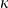
B, two for INCY and one for SOCS-1 respectively. The sink (0,0,0,0,0) represents the normal behavior where inflammation is produced in response to infection and the system reverts to this resting state after mounting an inflammatory response. In contrast, the sink (0,0,1,2,1) represents a diseased behavior where INCY levels are very high and a condition of hyperinflammation is produced. MAL state space allows for transitions between different states forming trajectories or paths that lead toward either the (0,0,0,0,0) or the (0,0,1,2,1) sink and the significance of these transitions is that they represent the indigenous ability of the system to attain stability.

The complete state space is very large and it becomes important to isolate key trajectories that lead either to a normal or diseased sink. The shortest possible route toward the normal sink was identified and used as the reference trajectory (Figure 4). Small divergences from this trajectory that culminate at the normal state were also identified. Other critical divergences were those that lead to the diseased state and these present the perturbations the system may encounter. Divergences that eventually lead to the normal state indicate perturbations that the system can tolerate, while divergences that can only lead to the diseased state are perturbations that put too much stress on the system resulting in a collapse of the system controls.

This MAL state space suggests that the system maintains the capacity to stabilize itself after perturbations as long as the concentration of INCY for any particular state within the system remains at level 1. But once the concentration of INCY attains level 2, the system enters trajectories that lead only to the sink (0,0,1,2,1), representing the diseased behavior. It is important to note that the threshold concentration level for INCY for the diseased state is 2. This indication is supported by the experimental observations made in the case of bacterial sepsis where the pathogenesis is associated with the over production of INCY and hyperinflammation [40], [41]. The pathogenesis and tissue injury in case of sepsis is closely related to the severe malarial pathogenesis [42].

Another important indication is that the moment INCY appears, the system is at risk of entering trajectories that may only lead to the diseased state (Figure 4, transitions 23 and 24). For the normal trajectory the INCY concentration level is maintained at 1, however when concentration level of 2 for INCY is acquired, the system loses its capacity to revert toward normality and moves towards the diseased state in a progressive manner. Furthermore, since INCY are produced through several alternate pathways, the model suggests that if INCY levels are already elevated due to some other reason, like prior inflammatory condition, the chance of the system shifting towards diseased state elevates. The state (0,0,1,1,1) is indicative of this where it culminates directly at the diseased state with only one transition (Figure 4, transition 24). Moreover, the model also indicates that an important component for this BRN (apart from MAL) is BTK which remains constitutively active in the cases where the divergence from the normal trajectory leads to the diseased state (Figure 4, for example the trajectory with transitions 1, 10, 12, 14, 22, 18 and 19). BTK is important for activation of MAL through phosphorylation, and this suggests that BTK may also play an important role in pushing the system towards hyperinflammation, and warrants further investigation.

### Hybrid Modeling

The rate at which a component of a BRN is regulated in relation to other components determines the direction of the evolution of the system. These rates are proportional to the threshold concentrations for each component and are modeled as unvalued parameters with delays. HyTech [27] uses these delays as unvalued parameters along with the discrete model of our BRN to calculate the conditions, in the form of constraints that are required for a transition between two states to occur. A path in the model is a sequence of states leading to a particular stable steady state and HyTech synthesizes the conditions for transitions between the states (Table 1). The conjunctions of all the constraints of the path constitute the conditions for that path to be followed (Table 2). Only when these conditions are satisfied, the system will reach a stable state representing either normal or diseased behavior.

(1,1,1,1,0) 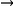 (1,1,1,2,0) & (0,1,1,1,0) 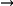 (0,1,1,2,0) (transitions 16 & 23): These transitions require that the rate of production of INCY in order to reach high level (level 2) should be faster than the induction of SOCS-1, suggesting that reducing high cytokine levels and inducing SOCS-1 can help to avoid this transition.

(1,1,1,1,1) 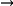 (1,1,1,2,1) (transition 22): The conditions for this transition suggest that if BTK remains active for a long period of time, the system will lead to the diseased state. This can be due to continuous activation of MAL by BTK and adapting strategies to prevent this is suggested.

(0,1,1,1,1) 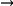 (0,1,1,2,1) & (0,0,1,1,1) 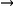 (0,0,1,2,1) (transitions 26 & 24): These transitions suggests that the system may also move towards diseased state due to multiple factors being involved that include: high rate of production of INCY; slow rate of deactivation of MAL, induction of SOCS-1, and deactivation of NF-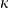
B. To avoid these transitions, intervention at multiple levels is suggested that include: reducing high cytokine levels, NF-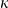
B and their effects; faster rate of deactivation of MAL which in turn is affected by higher rate of SOCS-1 induction; slower rate of activation of MAL being controlled by BTK.

The conditions (Table 1) for transition between these states imply that, to avoid hyperinflammation, strategies should be designed that: i) inhibit continuous activation of MAL by BTK, ii) reduce the effect of high cytokine levels and iii) induce SOCS-1. Our results suggest that under certain conditions, MAL related inflammatory response in malaria patients, may cause hyperinflammation leading to CM. We hypothesize that constitutively active BTK keeps activating MAL to produce the diseased behavior and may be an important drug target. BTK, a tyrosine kinase enzyme, is an attractive drug target and inhibitors are already being developed against it for a number of diseases like B-cell lymphomas and some autoimmune disorders [43].

The rate at which INCY are produced within the system is crucial and will determine its fate [44]. These key divergence states have INCY at level 1 and the system is at risk of going into the diseased state if these levels are further increased. Individuals can have high INCY levels due to various reasons which may include genetic predisposition, repeated exposure to infectious agents or chronic inflammatory conditions. Such conditions may partially explain prevalence of CM in some Sub Saharan African populations where malaria is endemic all year round and cytokine levels are high due to asymptomatic parasitaemia [45]–[49]. The effects of high cytokine levels may be controlled through the use of anti-inflammatory agents. A recent study by Franklin et al., 2011 [50], emphasizes the relationship between elevated levels of inflammatory cytokines and CM. They report a promising strategy for the management of malarial severity by interfering with the release of cytokines through inactivation of TLRs using antagonists. This supports our second hypothesis that suggests an association between a preexisting high level of cytokines and the progression of malaria towards CM. We suggest that such individuals or population groups may be more susceptible to develop CM and this opens up new avenues for future investigations.

### Conclusions

The normal (inflammatory) and abnormal behaviors (hyperinflammatory), presented by this qualitative model are consistent with experimental observations. They are commonly observed among population groups where in some the malarial infection is cleared after a normal inflammatory response, while in others severe malaria (CM) may develop due to hyperinflammation.

This study suggests an association between pre-existing high cytokine levels and progression of malaria towards CM, and opens up new avenues of investigation. This means that screening population groups, in areas where CM is endemic, for inflammatory cytokine levels may help identify ‘at risk’ individuals. This can be used as a predictive indicator that such an individual may progress towards CM. Multiple strategies that interfere with the TLR signal transduction, e.g. the use of BTK inhibitors and antinflammatory agents along with existing treatments should be further explored.

This study also shows that formal methods can support BRN modeling, which in turn facilitates state space exploration and trajectory computation. In future we plan to conduct the formal probabilistic analysis [51] to gain more insight into the MAL signaling network.

## Materials and Methods

### Qualitative modeling

Biological systems are traditionally modelled with ordinary differential equations that refer to the time derivative of each quantity (concentration, rates and temperature etc.). Due to the inherent complexity of biological systems, the exact amounts of these quantities are rarely known. In 1970, René Thomas [38] introduced a Boolean logic based method for the modeling of biological regulatory networks in which the system dynamics are modelled qualitatively. He proved the effectiveness of his modeling approach by analyzing the lambda phage genetic switch [38], [52]–[55]. According to René Thomas, the Boolean modeling suffers from some limitations because it has only two levels, 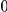 or 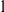, which are not sufficient to formalize all kinds of relevant problems. Later on, Thomas generalized the Boolean logic to ‘kinetic logic’ [28]–[36] and showed its practical effectiveness by applying it to different genetic regulatory systems. An interesting feature of ‘kinetic logic’ is that it closely approximates the differential equations based modeling [29].

### The Kinetic Logic formalism of René Thomas

The kinetic logic formalism models BRNs by focussing on threshold effects. The entities (usually proteins) of biological systems can interact with each other either positively or negatively; that is the concentration level of one element can increase or decrease the rate of activation of other elements. In our setting activation means production of a particular protein, if it is absent, or its activation from an inactive state. Similarly, inactivation means inhibition of a particular protein or its degradation. Following are the two types of biological regulation which are of sigmoid (nonlinear bounded curve) nature.

### Activation

**Figure 5:**
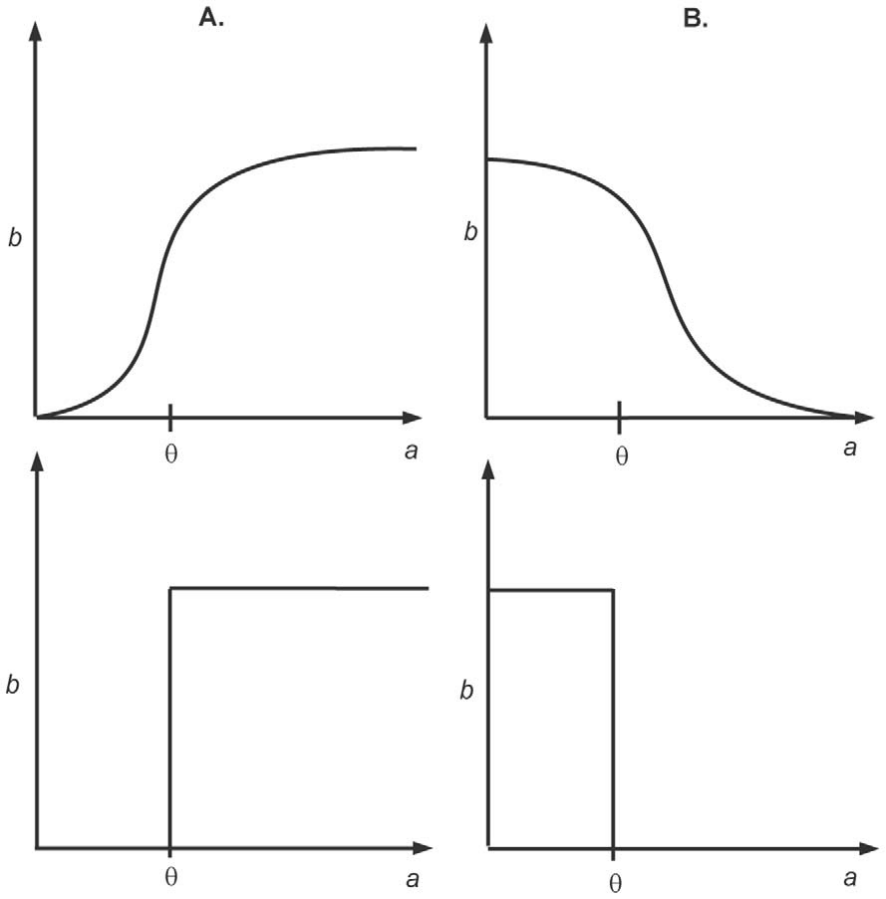
Activation and degradation of entities. (A.) Sigmoidal (above) and Step function (below) representation of activation. (B.) Sigmoidal (above) and Step function (below) representation of degradation (inhibition).

If a product 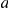 increases the rate of activation of product 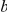, it is a positive regulator. In this situation the rate of activation of 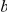 increases with increasing concentration of 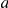 , which can be depicted by a sigmoidal representation as shown in Figure 5. It can be seen that there is little effect of 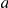 on the rate of activation of 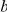 for as long as it remains below a given threshold, 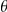. Once 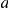 reaches this threshold, the rate of activation of 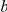 increases rapidly until 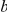 is saturated. In other words we can say that 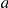 was ‘inactive’ when 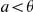 and ‘active’ for 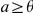, this suggests the approximation of the sigmoidal curve by a step function as shown in Figure 5(A.).

### Inhibition

If product 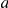 reduces the rate of activation of product 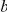, it is called a negative regulator or inhibitor. The effect of this negative regulation or inhibition is shown in Figure 5(B.).

### Semantics of the Kinetic Logic Formalism

This section presents the semantics of Thomas's formalism with a hypothetical running example of a gene regulatory network involving three genes (x, y and z).

**Definition 1 (Directed Graph).** *A directed graph is an ordered pair* 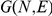 *, where*

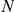
 is the set of all nodes and

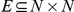
 is the set of ordered pairs called edges or arcs

An edge 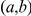 is considered to be directed from 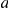 to 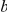; 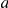 is called the head and 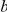 is called the tail of the edge. In a directed graph 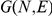, 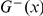 and 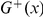 denote the set of predecessors and successors of a node 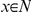, respectively.

**Figure 6:**
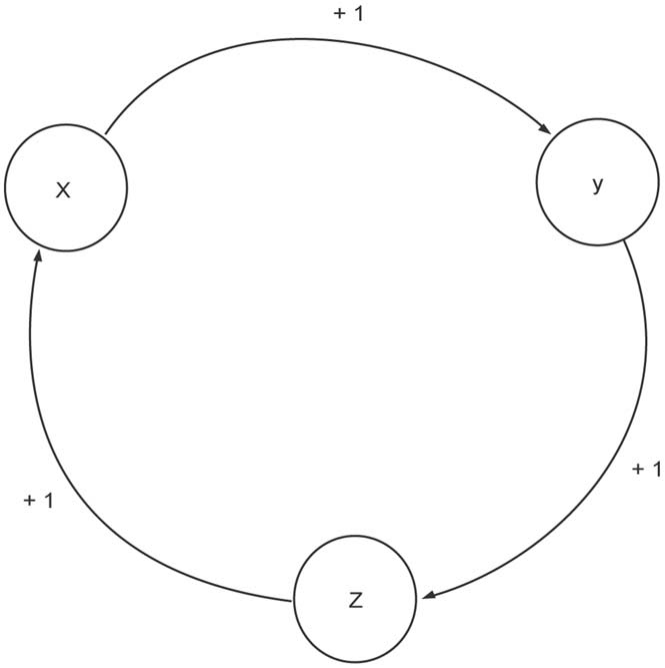
A toy BRN. A toy example of a BRN where 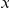, 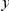 and 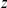 represent biological entities. The labels ‘+’ and ‘1’ represent the activation and the threshold concentration respectively.

Figure 6 shows a network represented by a directed graph showing the genes in which 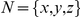 and 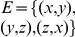 are the sets of nodes and edges, respectively. In case of gene 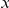, (The set of regulators of 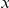) 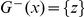 and 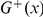 = 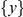 represents the predecessors and successors of 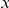.

**Definition 2 (Biological Regulatory Network).** *A biological regulatory network (BRN) is a labeled directed graph* 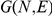 *, where* 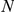 *is a set of nodes which represents biological entities and* 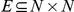 *, is a set of all possible edges which represent the interaction between entities.*

- Each edge 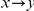 is labeled by a pair 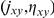, where 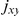 is positive integer (qualitative level representing a threshold) and 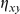 is either the (+) sign or the (−) sign, where (+) represents activation and (−) inhibition. For an example see Figure 6, where 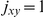, 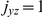,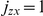 and similarly 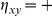, 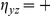, 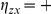.
- Each node 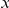 has a limit 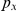, which is equal to out-degree of 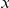 (the total number of targets of x), such that 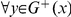 each 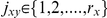 where 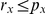.
- Each entity 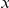 has its abstract concentration in the set 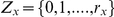.

It is important to consider the possible number of states and transitions between them to understand the behavior of the BRN.

**Definition 3 (States).** *Let* 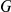 *be a BRN. The state of a BRN is a tuple* 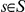 *, where*

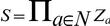



The qualitative states are represented by vector 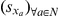, where 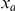 denotes the level of concentration of product 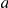. A qualitative state represents a configuration of all the elements of a BRN at any instant of time. The number of activators of a particular variable at a given level of concentration are represented by its set of resources (see the definition of Resources given below). In the following definition, we formally define the set of resources which represents all the activators of an entity at any instance of time.

**Definition 4 (Resources).** *Let* 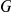 *be a BRN. The set of resources* 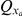 *of a variable* 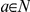 *at a level* 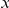 *is defined as* 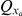 *=* 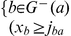 *and* 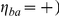 *or* 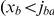 *and* 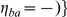 *.*

The dynamic behaviors of BRN depends on logical parameters. The set of these logical parameters is defined as 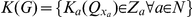.

The parameter 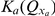 (at a level 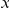 of 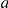) gives the information about the evolution of 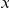. There are three cases: 1) if 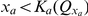 then 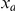 increases by one unit, 2) if 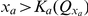 then 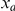 decreases by one unit, and 3), if 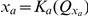 then 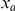 cannot evolve from its current level. In case of the running example of three genes (Figure 6), the set of resources and logical parameters are shown in Table 3.

**Table 3:** Table of states, resources and logical parameters.

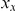	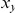	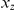	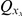	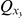	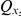	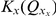	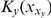	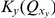
0	0	0	–}	–}	–}	0	0	0
0	0	1	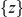	–}	–}	1	0	0
0	1	0	–}	–}	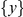	0	0	1
0	1	1	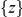	–}	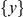	1	0	1
1	0	0	–}	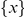	–}	0	1	0
1	0	1	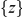	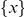	–}	1	1	0
1	1	0	–}	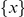	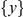	0	1	1
1	1	1	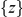	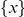	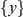	1	1	1

This table lists all the states, set of resources and logical parameters of the BRN in Figure 2.

It is convenient to describe the evolution from one level to the other by an evolution operator  <$>\raster="rg1"<$> [56], which is defined as follows:
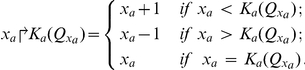
where 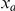 and 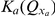 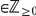


**Definition 5 (State Graph).** *Let* 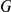 *be a BRN and* 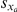 *represents the concentration level of entity* 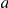 *in a state* 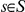 *. Then the state graph of the BRN will be the directed graph* 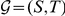 *, where* 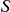 *is set of states and* 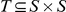 *represents a relation between states, called the transition relation, such that* 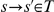 *if and only if:*

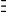
 a unique 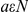 such that 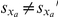 and 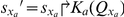, and

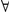
 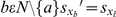.

**Figure 7:**
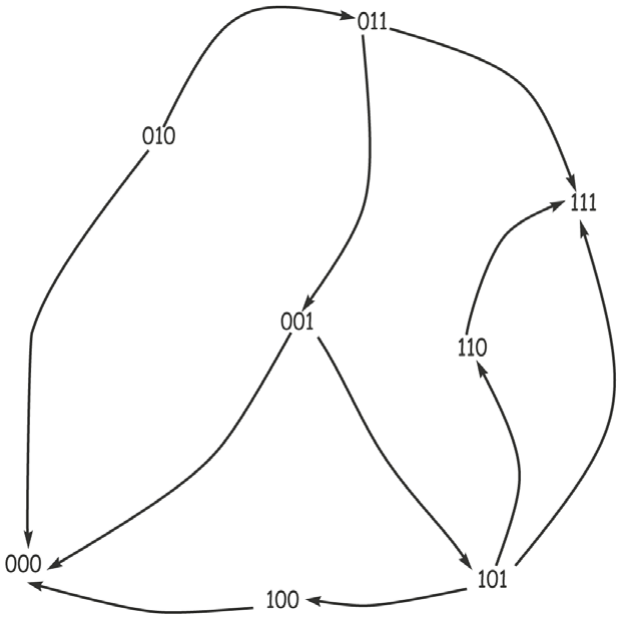
State graph. The state graph of the BRN in Figure 3. Each node represents a qualitative (discrete) state of the BRN. The values inside a state represent the concentrations of the entities 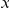, 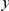 and 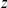.

From the table of resources (Table 3), interesting information regarding its dynamic behavior can be derived, c.f. Figure 7. It is clear from the state graph that there are two stable states, namely (000) and (111).

### Discrete Modeling of MAL associated BRN using GENOTECH

GENOTECH [26] facilitates modeling of BRNs according to Thomas' formalism. It takes a BRN and the corresponding logical parameters form a user in a simple graphical user interface (GUI). It automatically produces the whole state space in which stable states, cycles and paths between any two states can be identified. The software tool GINsim (Gene Interaction Network simulation) [57] could also be used for the qualitative modeling and analysis of BRNs. GENOTECH is available for download at http://code.google.com/p/genotech/downloads/list and the following step are required for modeling a BRN in GENOTECH.

**Figure 8:**
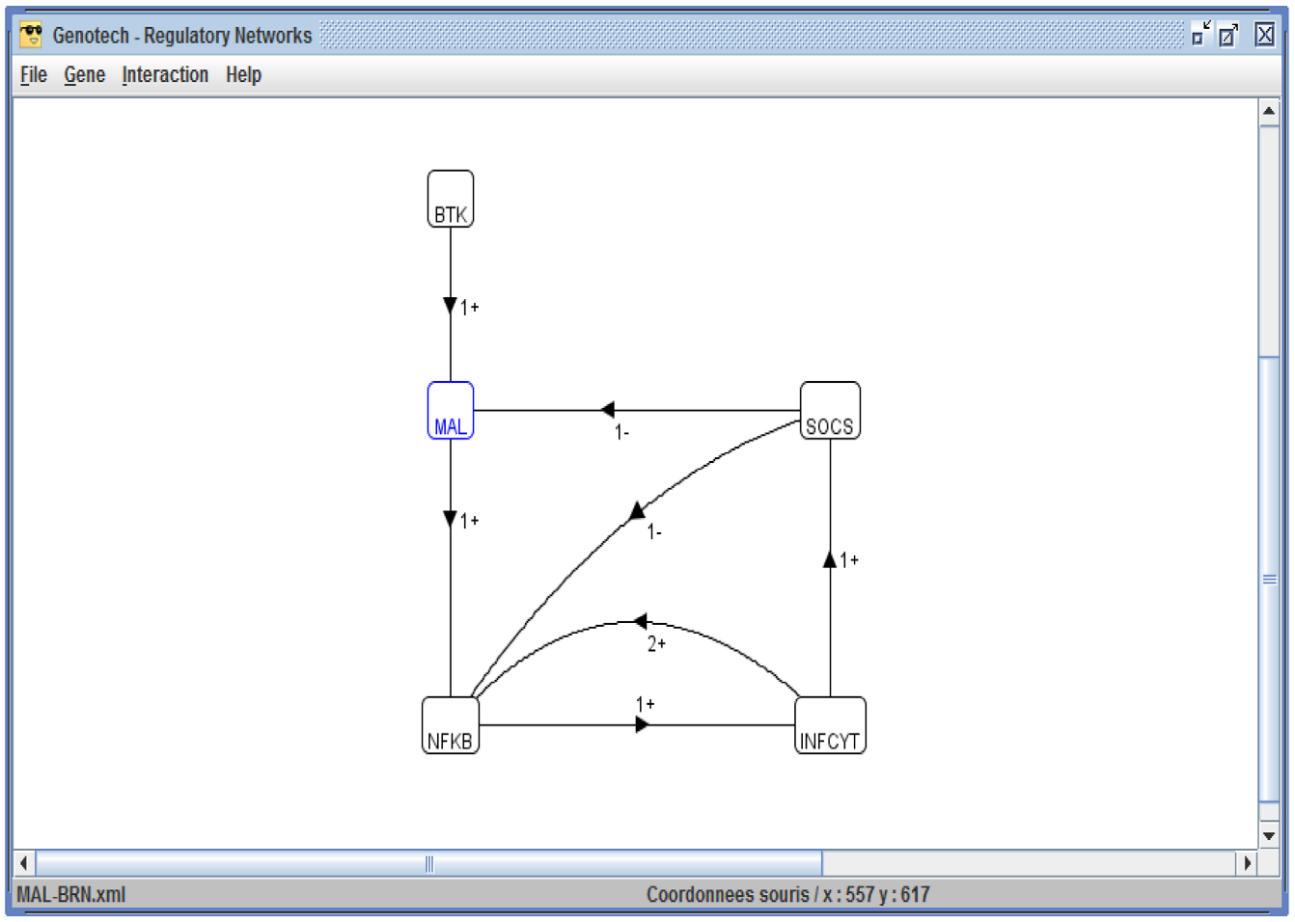
Snapshot of the MAL associated BRN construction in GENOTECH. The BRN is constructed as a directed graph by using the Gene’ New and Interaction’New menu options. An edge between two entities shows an interaction which is labeled with threshold and the sign of interaction (+for activation and −for degradation).

**Figure 9:**
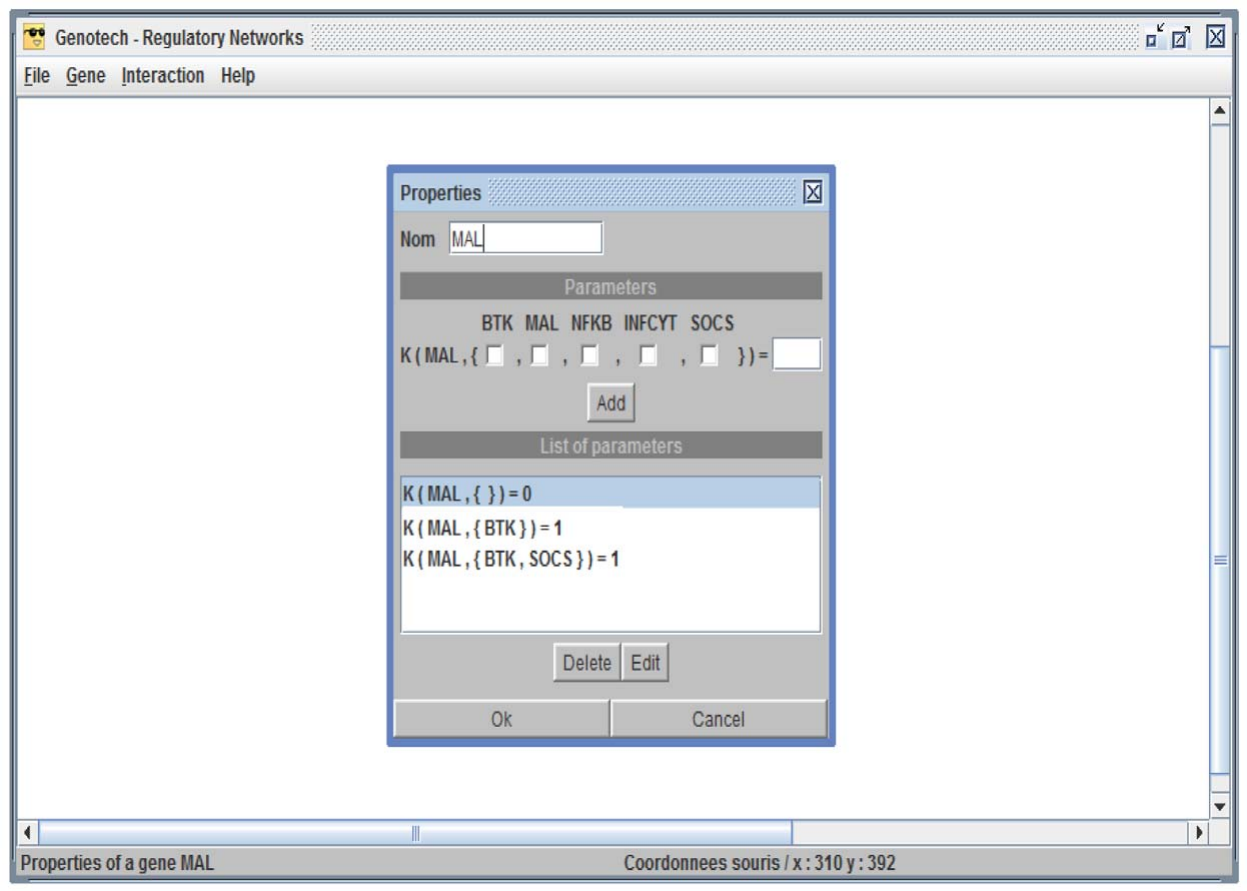
Snapshot of the logical parameters. Each entity of the BRN is assigned a set of logical parameters by using the properties option accessible by right clicking that entity.

**Figure 10:**
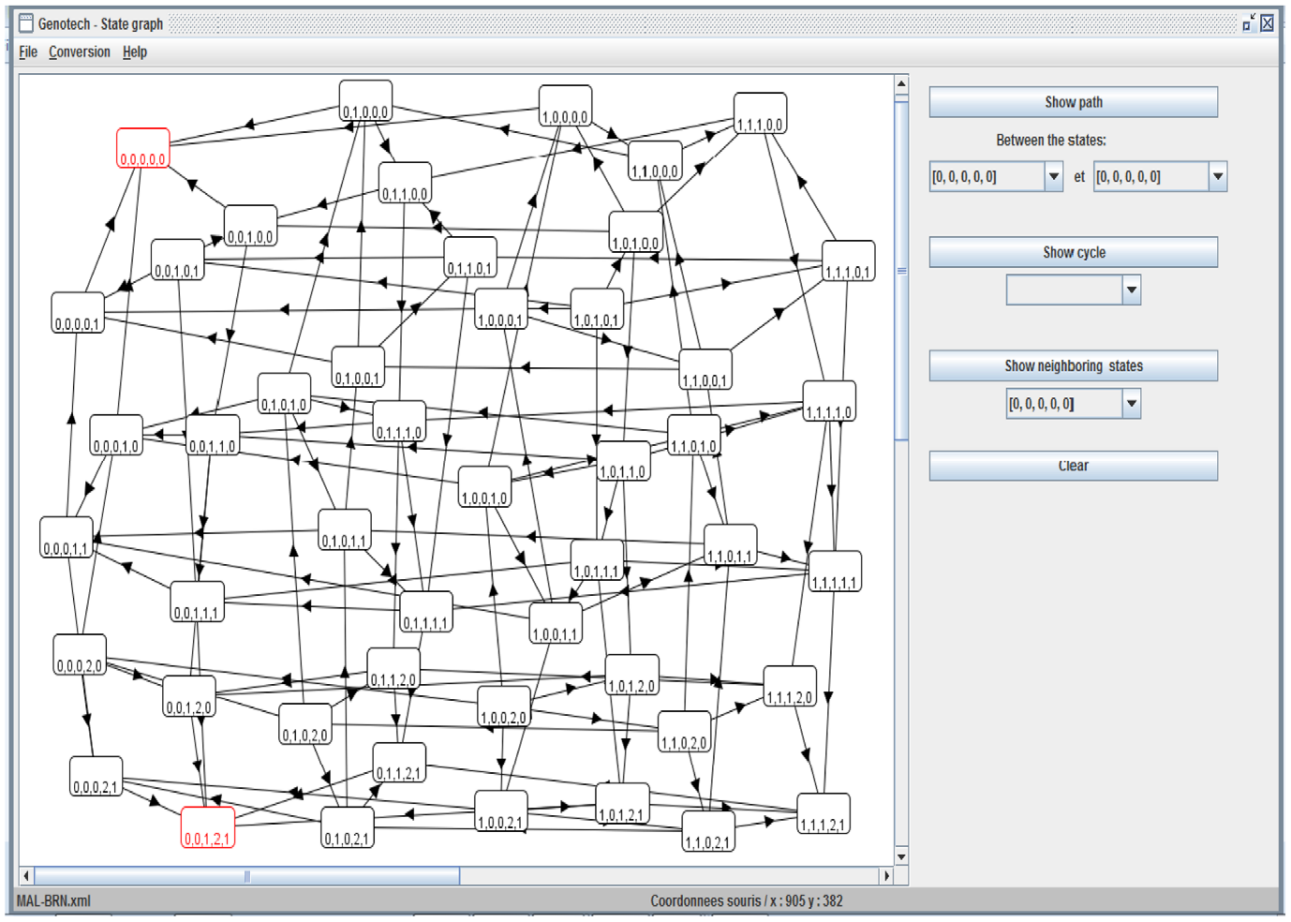
Snapshot of the state graph in GENOTECH. The stable states are highlighted in red. The right panel shows the analysis command which include: show path which highlights paths between two selected states; show cycle which highlights existing cycles (closed path), if any, in the state graph; show neighboring states highlights all the neighboring states of the selected state. The conversion menu contains the commands to export the graph to DOT and HyTech formats.

1. Construction of a BRN as a labeled directed graph: The GUI of GENOTECH provides a set of commands to create, edit and save a BRN. The two drop down menus Gene and Interaction are used to construct the nodes and edges of a BRN respectively; and each biological entity is assigned a set of logical parameters (see Figure 8 and 9).
2. Generation of a state graph: After the construction of the BRN, a state graph can be generated by using the command state graph in the File menu. The state graph, showing stable states in red, appears in a new window with its own set of commands to find cycles, paths between two states, and neighboring states (see Figure 10). GENOTECH also provides an option to save the state graph in DOT format [58] for visualization in Graphviz tool [59].

The complexity of a state graph increases depending on the number of entities, their interactions, thresholds and logical parameters. In order to make this method more scalable, a simplification technique based on the coloration method, which extracts the desirable parts of the state graph, has been proposed in our previous work [60] – the future versions of GENOTECH will implement this technique.

Figure 1 shows the TLR2/4 molecular pathway adapted from literature [15], [24], [25]. The MAL associated BRN was abstracted from this pathway and is shown in Figure 2. The Network flow is as follows: BTK activates MAL and MAL activates NF-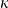
B after reaching threshold level of 1; INCY are activated by NF-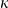
B at a threshold level of 1; there are two interactions of INCY which are the activations of SOCS-1 and NF-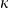
B at level 1 and level 2 respectively; and SOCS-1 inhibits MAL and NF-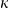
B at threshold level 1. Using the Thomas' formalism presented in the previous section, preliminary insilico experiments were performed to set the threshold orders and to determine the values of logical parameters (see Figure 3) in order to make the model consistent with naturally observed behaviors (steady states).

### Hybrid modeling to enhance the discrete modeling with delays

**Figure 11:**
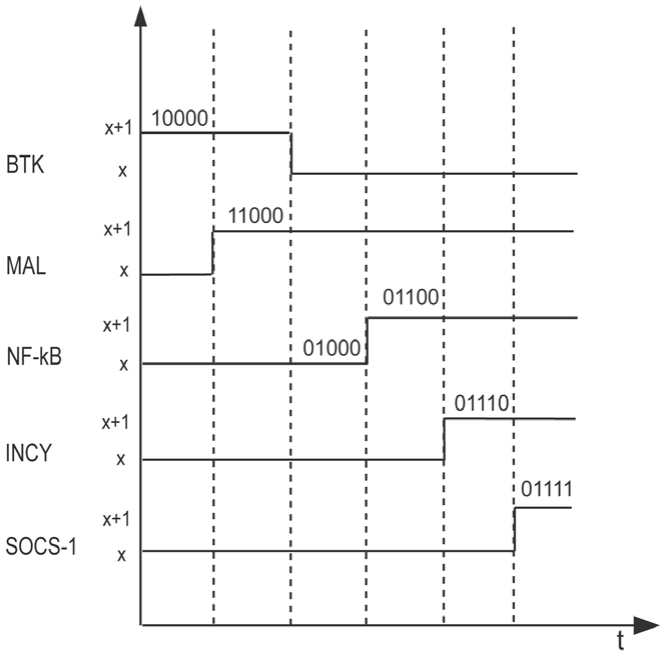
Timing diagram. Timing diagram showing the evolution of proteins involved in BRN. Here the concentrations are represented by two levels x and x+1 on the vertical axis. The horizontal axis represent the time of evolution. The dotted lines are the boundaries of the different configurations of the discrete concentrations. Each configuration is according to the order (BTK , MAL, NF-kB, INCY, SOCS-1).

**Figure 12:**
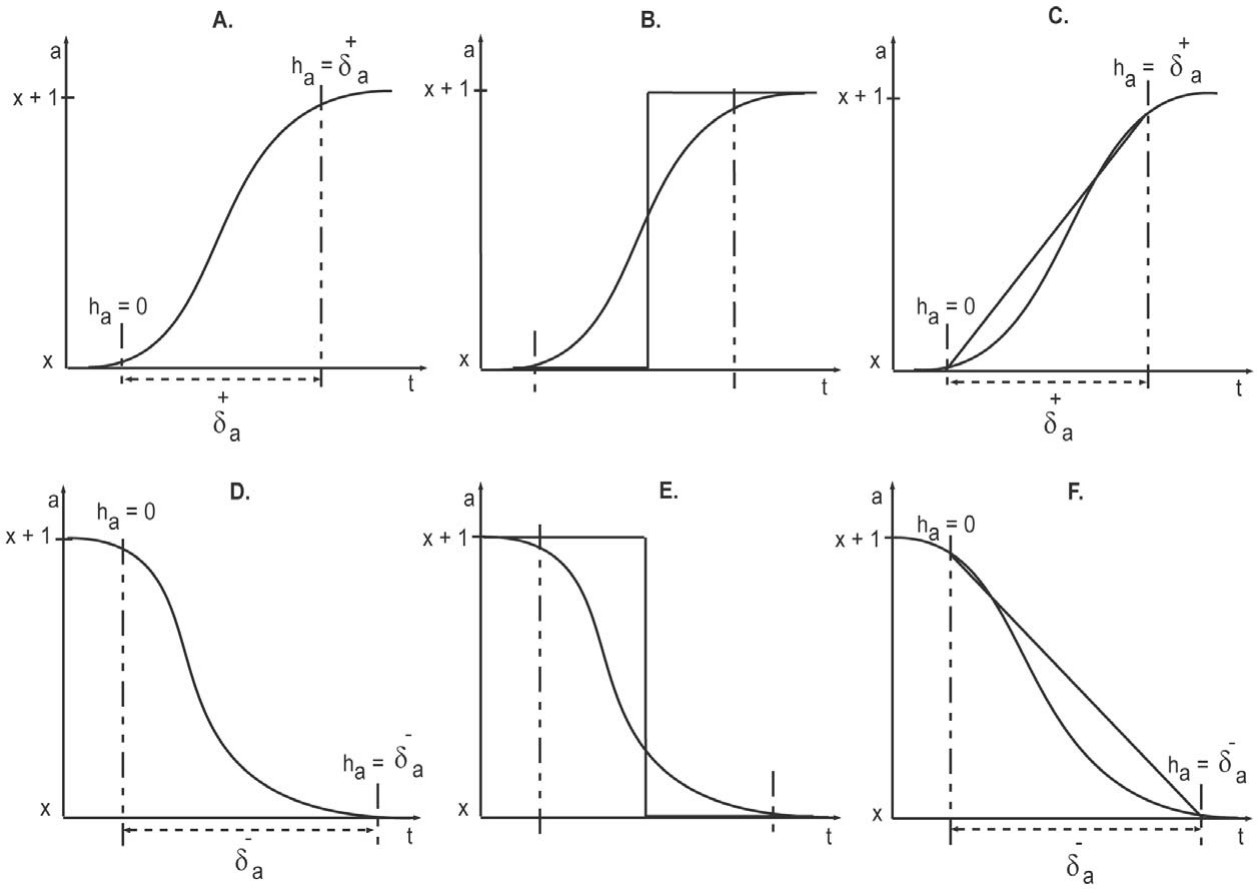
Different representations of the evolution of an entity. Evolutions of an entity 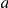 is shown as: sigmoidal representation of the activation (A.), discrete approximation of the activation (B.), piece-wise linear approximation of the activation (C.), sigmoidal representation of the degradation (D.), discrete approximation of the degradation (E.) and piece-wise linear approximation of the degradation (F.).

A hybrid model of a BRN involves discrete locations associated with some continuous variables (clocks). The continuous transitions represent time that elapses in a location, whereas discrete transitions show the instantaneous change between locations [61]. The timing diagram of one normal path is shown in Figure 11. It depicts proteins evolving from one level to another (from x to x+1 or x+1 to x) in a discrete fashion (Figure 12). In reality, however, the concentrations of these proteins evolve in a nonlinear and continuous manner and this behavior cannot be represented in a discrete modeling framework. Various formalisms [62], [63] have been proposed for biological modeling to overcome this limitation of discrete modeling. Ahmad et al. [64] proposed the refinement of discrete modeling by hybrid modeling, where sigmoidal nature evolutions are modelled with piecewise linear curves. This contrasts discrete modeling, in which these evolutions are modelled using step functions (Figure 12). Considering that a delay is required for the evolution of a protein from level 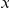 to 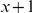, or from 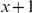 to 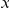, it is important to introduce some additional concepts, namely time intervals and clocks.

The approach of Siebert and Bockmayr [63] is similar to our approach. The authors incorporate time delays in the logical formalism of René Thomas using timed automaton [65] which is a more restrictively expressive model than hybrid automaton, since it does not deal with non-increasing variables.

Clocks are continuous variables used in timed automaton based models [65], which are a subclass of hybrid automata [66]. Each protein is associated with a clock variable 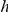 that synchronously evolves with time. These clock intervals reflect the characteristics of continuous dynamics within the available discrete formalism [61], [64]. The time measured by the clock variable 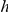 between two levels is called the delay between these levels. The initial value of the clock variable is set to zero and when the value of this clock variable is equal to delay time 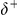 or 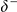 the transition between two levels takes place. The delays 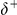 or 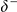 represents time taken from 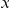 to 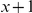 (positive delay or production delay) or 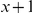 to 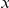 (negative delay or degradation delay), respectively (Figure 12). The speed by which the clock variables 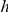 evolve is modelled by the first order derivatives 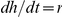, where 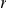 lies in the set 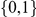. This value characterizes the evolution of the associated variable, which normally represents the concentration level of a protein [61], [64].

We thus obtain a hybrid model that is suitable to represent the discrete and continuous dynamics of the systems that we consider. In this work, delays are considered as unvalued parameters, which motivate the introduction of Parametric Bio Linear Hybrid Automata (Bio-LHA) for the modeling of the MAL associated BRN, as presented in the next subsection.

### Parametric Bio-LHA

Parametric Bio-LHA was originally proposed by Ahmad et al. [26] for the linear hybrid modeling of BRN. This framework refines the discrete (qualitative) model by incorporating clocks and time delays in it. The resultant model is then suitable for the computation of exact conditions in the form of delay constraints for the existence of the behaviors of BRN.

Let 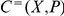 (resp. 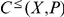) be the set of constraints using only 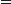 (resp. 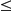) where 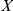 and 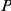 are the sets of real valued variables and parameters respectively.

**Definition 6 (Parametric Bio-LHA).** *A parametric Bio-LHA* 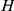 *is a tuple* 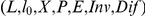 *where*

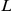
 is a finite set of locations.

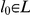
 is the initial location.

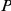
 is a finite set of parameters (delays).

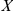
 is a finite set real-valued variables (clocks).

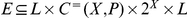
 is a finite set of *edges*, 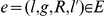 represents an edge from the location 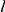 to the location 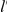 with the guard 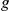 and the reset set 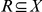; we require that the set of clocks in 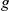 is a subset of 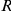.

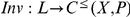
 assigns an invariant to any location.

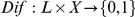
 maps each pair 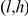 to an evolution rate.

The semantics of a parametric Bio-LHA is a timed transition system. We define the semantics according to the time domain 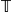. We let 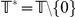.

**Definition 7 (Semantics of Bio-LHA).** *Let* 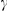 *be a valuation for the parameters* 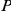 *and* 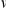 *represents the values of clocks in a location. The* 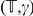 *-semantics of a parametric Bio-LHA* 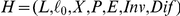 *is defined as a timed transition system* 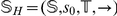 *where: (1)* 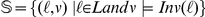 *; (2)* 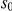 *is the initial state and (3) the relation* 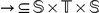 *is defined for* 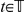 *as:*

- *discrete transitions:* 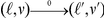 *iff* 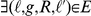 *such that* 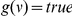 *,* 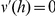 *if* 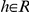 *and* 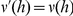 *if* 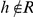 *.*
- *continuous transitions: For* 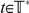 *,* 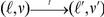 *iff* 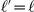 *,* 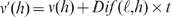 *, and for every* 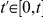 *,* 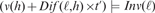 *.*

**Figure 13:**
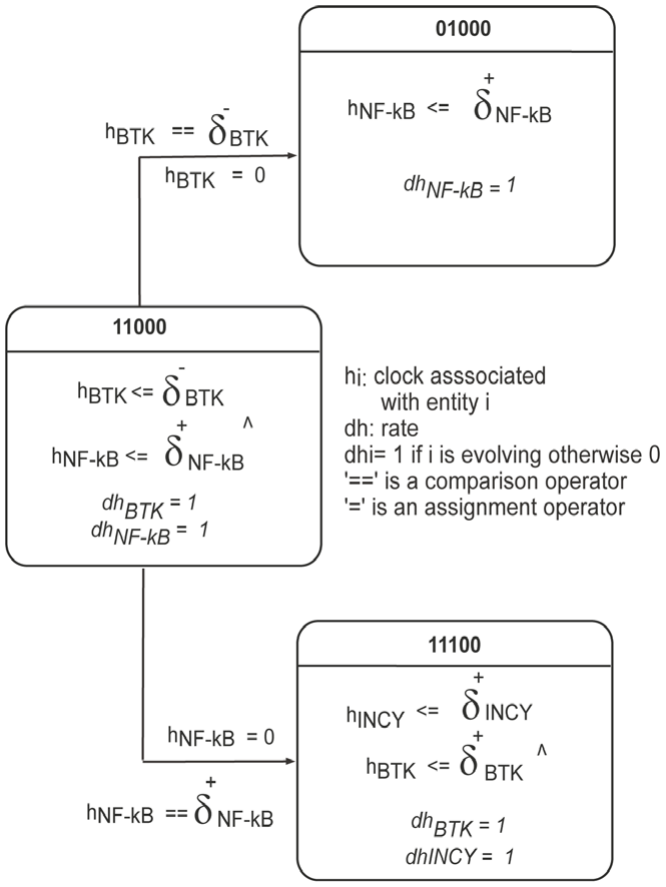
Bio-LHA. Partial view of Bio-LHA of the MAL associated BRN. The labels 11000, 01000 and 11100 shows the state (configuration) of BRN consisting of five entities (BTK, MAL, NF-kB, INCY, SOCS-1).

The partial Bio-LHA of the MAL associated BRN is shown in Figure 13. For the sake of simplicity, only three locations are considered in this model. The inequalities, such as (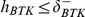), represent invariants or the stay conditions for the BRN to remain in a particular configuration (location). For example, (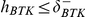) requires that the clock variable 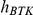 of BTK should be less than or equal to the degradation delay (time required to change level 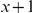 to 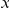 ) of BTK.

The rate dhBTK (HyTech representation of dhBTK/dt) provides an indication about the evolution from one level to another. The clock variables 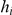 (i-th protein) measure the time taken by associated proteins in a particular level, and clocks are set to zero once the transition has occurred. The complete Bio-LHA involves forty eight locations and five clock variables associated with each protein involved in the BRN (Figure 2).

The next section presents the investigation of causality conditions for the normal and the divergent behaviors.

### Synthesis of delay parameters using HyTech: A Linear Hybrid Model checking approach

In this study, a Bio-LHA was used for the hybrid modeling of the MAL associated BRN. As discussed above, the unvalued delays represent parameters in the Bio-LHA. In order to analyze this model, the linear hybrid model checking tool HyTech was used, which is widely applied to the modeling and verification of hybrid systems. The main motivation for using HyTech for the analysis of this class of systems is its very rich set of analysis commands as well as the parameter synthesis capabilities of this tool (Table 1 and 2).
